# Supplementary material for: Connecting Epigenetic and Genetic Diversity of LTR Retrotransposons in Sunflower (Helianthus annuus L.) and Arabidopsis thaliana L
Source: Plants (Basel). 2026 Jan 9;15(2):204. doi: 10.3390/plants15020204 (PMC12844716; doi:10.3390/plants15020204)
Supplement: Supplementary file 1 [file plants-15-00204-s001.zip › manuscript-supplementary.pdf]

Supplementary material

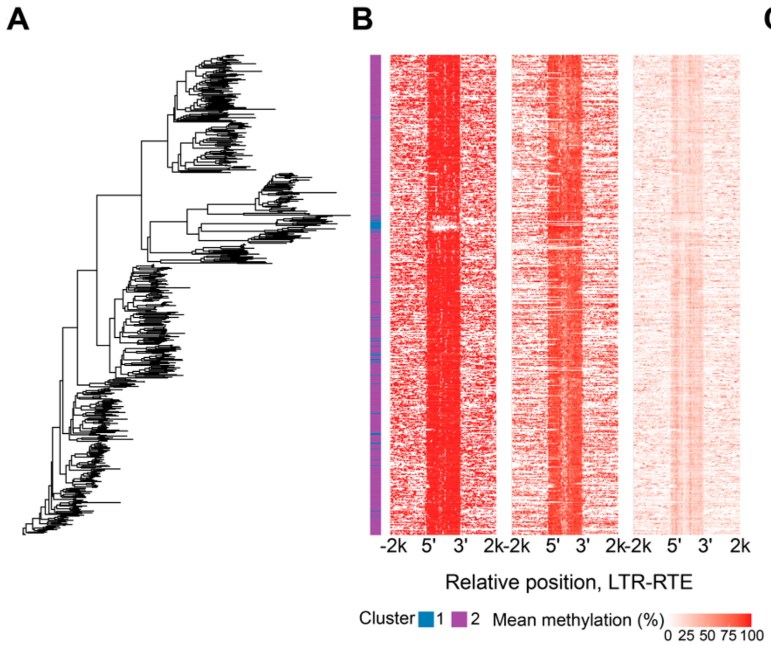

Supplementary Figure S1. Methylation patterns and phylogeny of *H. annuus Ale* LTR-RTE lineage. (A) Phylogenetic tree of reverse transcriptases from individual elements, constructed using the neighbor-joining method. (B) Heatplot of LTR-RTEs average methylation level across element length for CG, CHG and CHH contexts, rows represent individual elements grouped on clusters, where each row corresponds to a leaf on the phylogenetic tree. (C) Methylation pattern of individual clusters which show smoothed mean (solid line) and standard deviation (shaded area) of binned methylation.

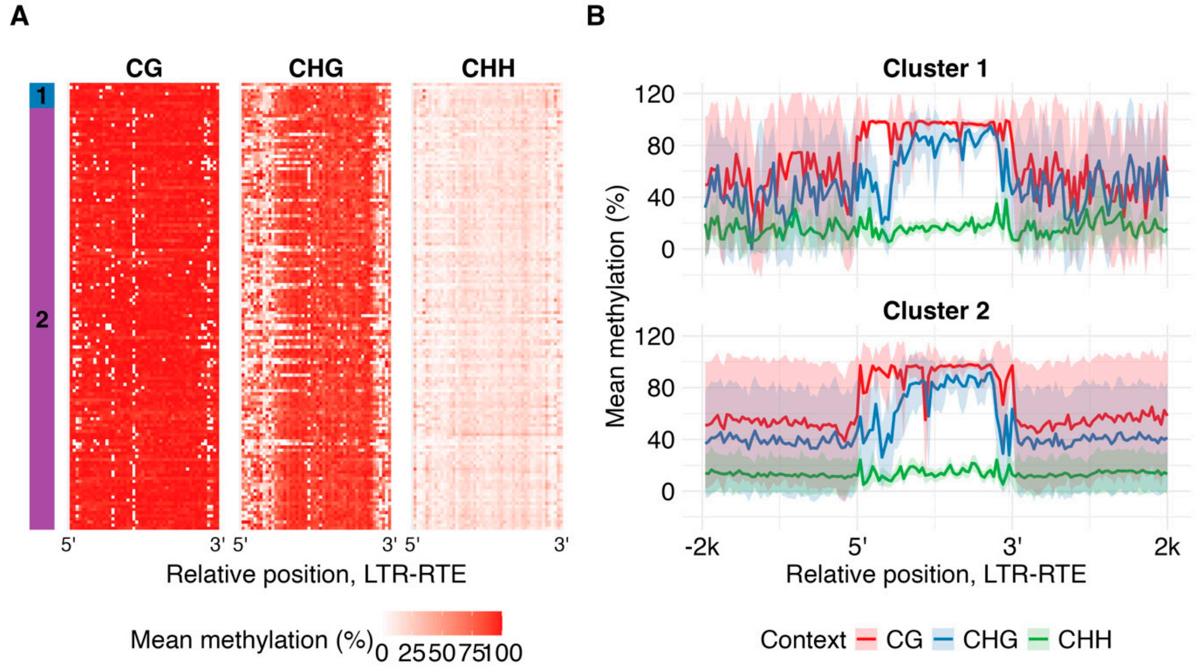

Supplementary Figure S2. Methylation patterns of *H. annuus Angela* LTR-RTE lineage. (A) Heatplot of LTR-RTEs average methylation level across element length for CG, CHG and CHH contexts, rows represent individual elements grouped on clusters, where each row corresponds to a leaf on the phylogenetic tree. (B) Methylation pattern of individual clusters which show smoothed mean (solid line) and standard deviation (shaded area) of binned methylation.

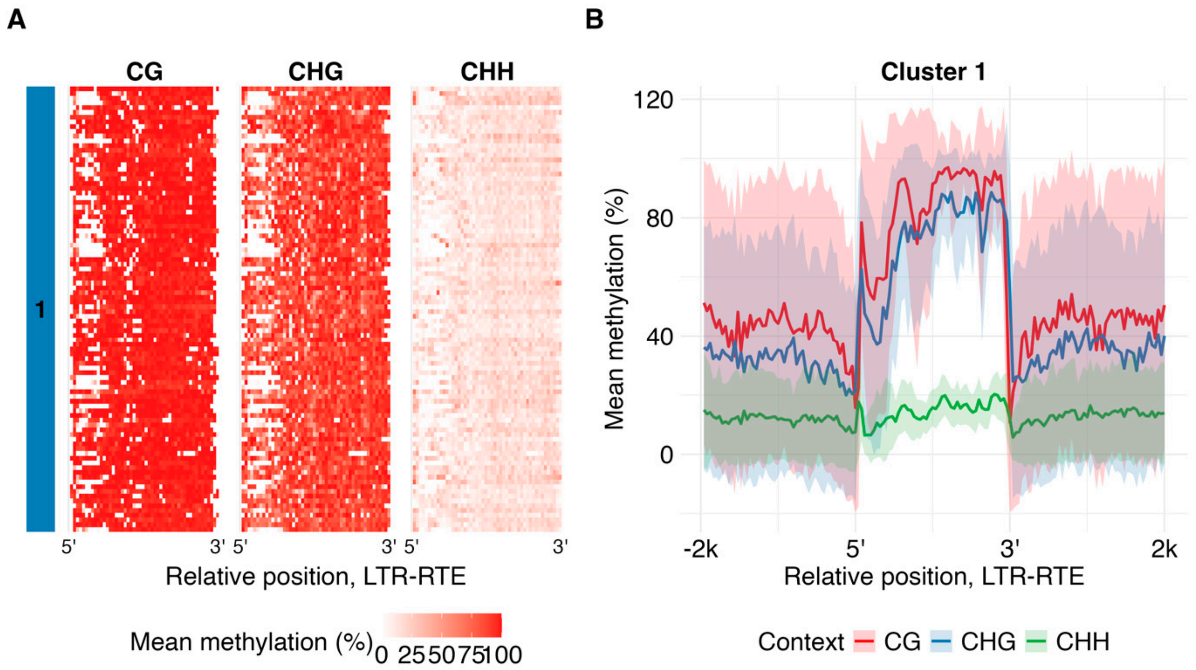

Supplementary Figure S3. Methylation patterns of *H. annuus Bianca* LTR-RTE lineage. (A) Heatplot of LTR-RTEs average methylation level across element length for CG, CHG and CHH contexts, rows represent individual elements grouped on clusters, where each row corresponds to a leaf on the phylogenetic tree. (B) Methylation pattern of individual clusters which show smoothed mean (solid line) and standard deviation (shaded area) of binned methylation.

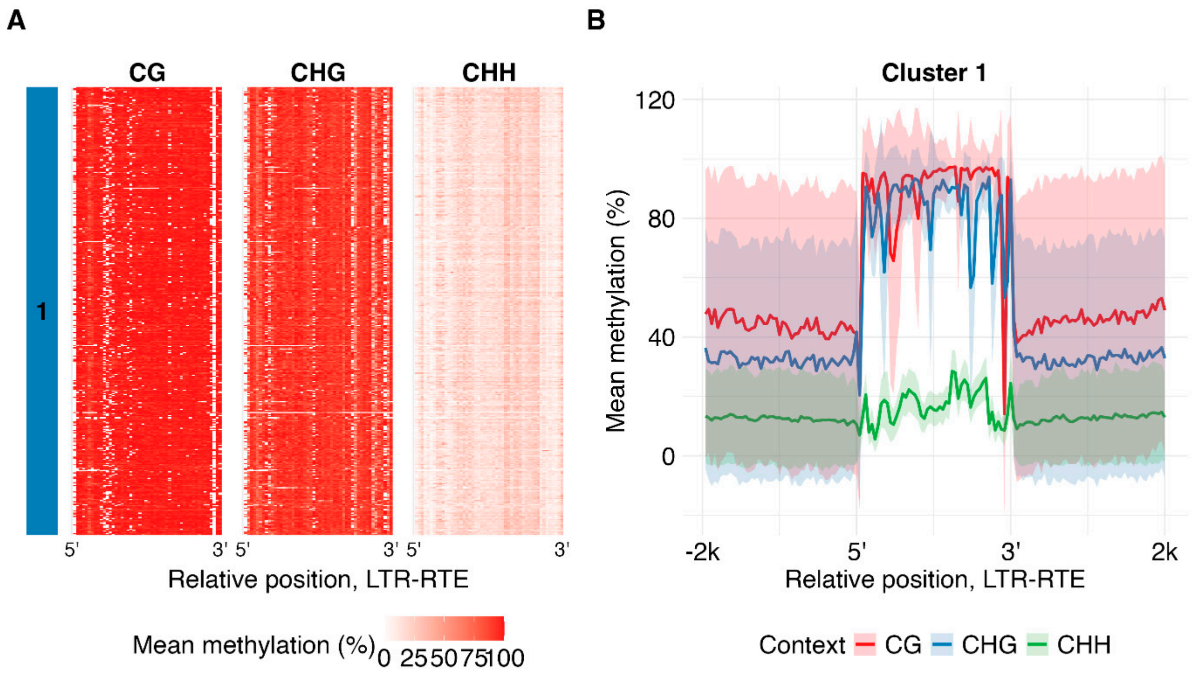

Supplementary Figure S4. Methylation patterns of *H. annuus Ikeros* LTR-RTE lineage. (A) Heatplot of LTR-RTEs average methylation level across element length for CG, CHG and CHH contexts, rows represent individual elements grouped on clusters, where each row corresponds to a leaf on the phylogenetic tree. (B) Methylation pattern of individual clusters which show smoothed mean (solid line) and standard deviation (shaded area) of binned methylation.

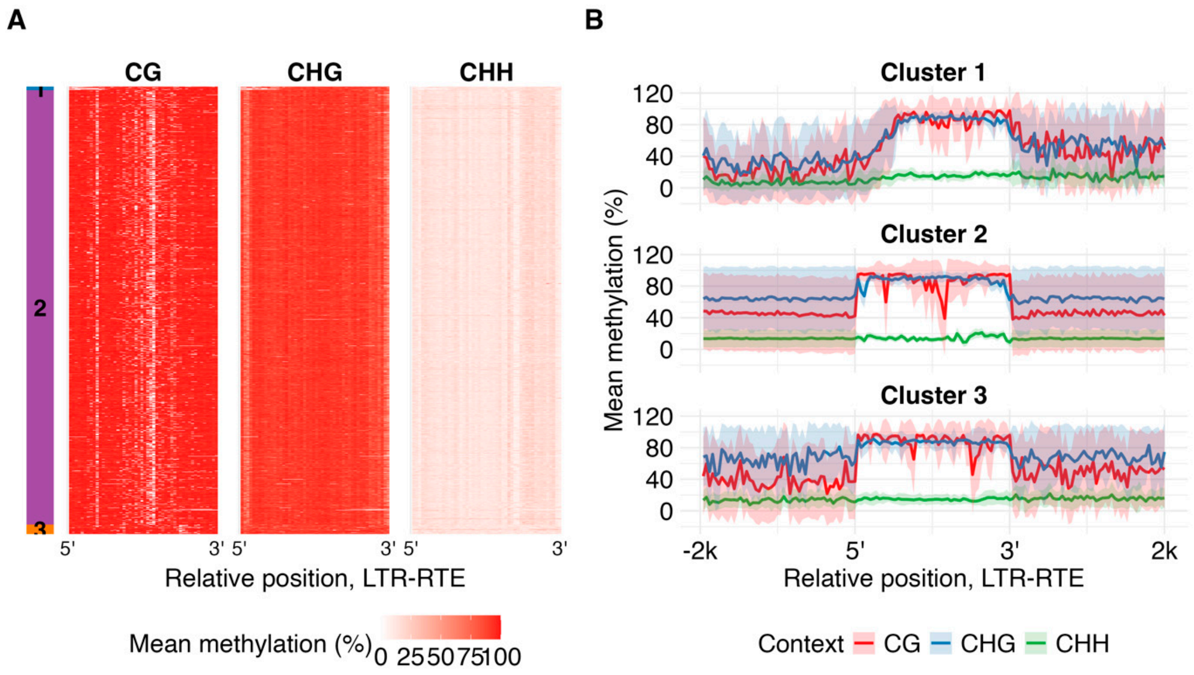

Supplementary Figure S5. Methylation patterns of *H. annuus Retand* LTR-RTE lineage. (A) Heatplot of LTR-RTEs average methylation level across element length for CG, CHG and

CHH contexts, rows represent individual elements grouped on clusters, where each row corresponds to a leaf on the phylogenetic tree. (B) Methylation pattern of individual clusters which show smoothed mean (solid line) and standard deviation (shaded area) of binned methylation.

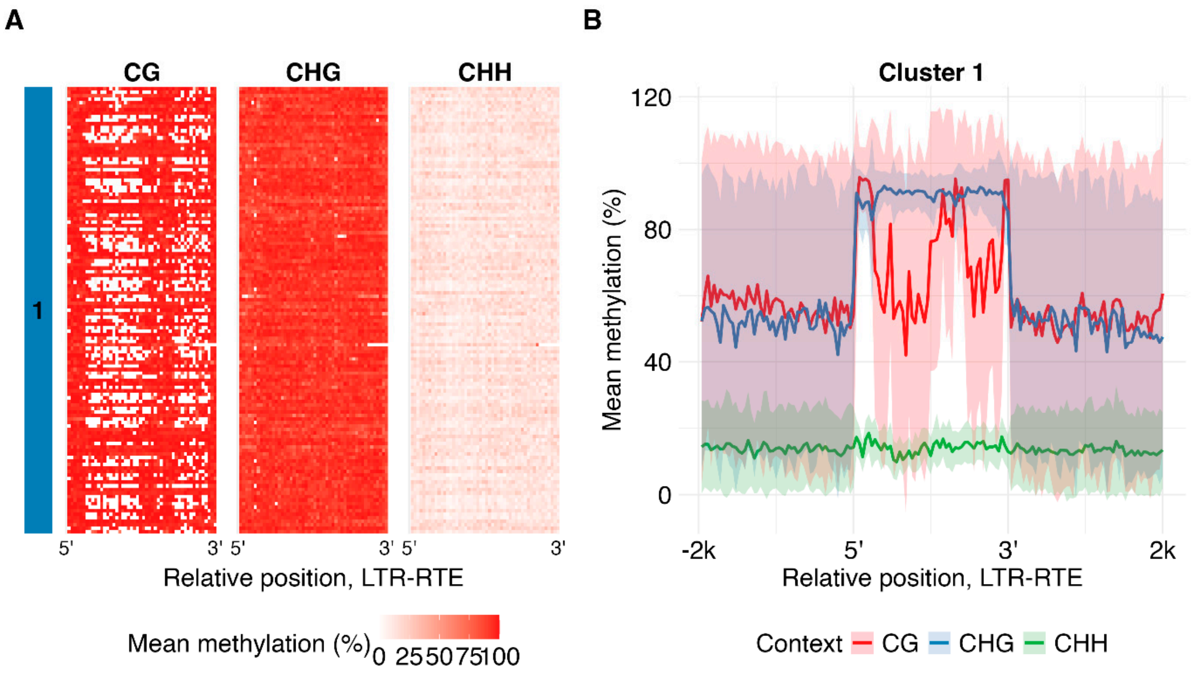

Supplementary Figure S6. Methylation patterns of *H. annuus SIRE* LTR-RTE lineage. (A) Heatplot of LTR-RTEs average methylation level across element length for CG, CHG and CHH contexts, rows represent individual elements grouped on clusters, where each row corresponds to a leaf on the phylogenetic tree. (B) Methylation pattern of individual clusters which show smoothed mean (solid line) and standard deviation (shaded area) of binned methylation.

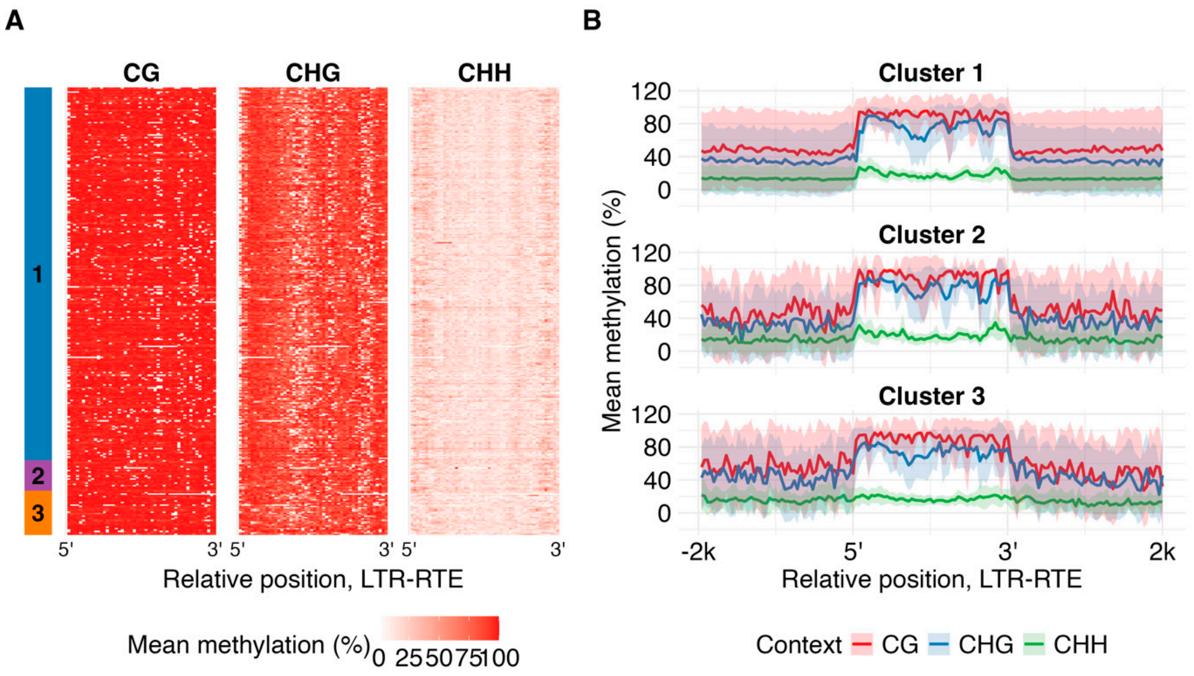

Supplementary Figure S7. Methylation patterns of *H. annuus TAR* LTR-RTE lineage. (A) Heatplot of LTR-RTEs average methylation level across element length for CG, CHG and CHH contexts, rows represent individual elements grouped on clusters, where each row corresponds to a leaf on the phylogenetic tree. (B) Methylation pattern of individual clusters which show smoothed mean (solid line) and standard deviation (shaded area) of binned methylation.

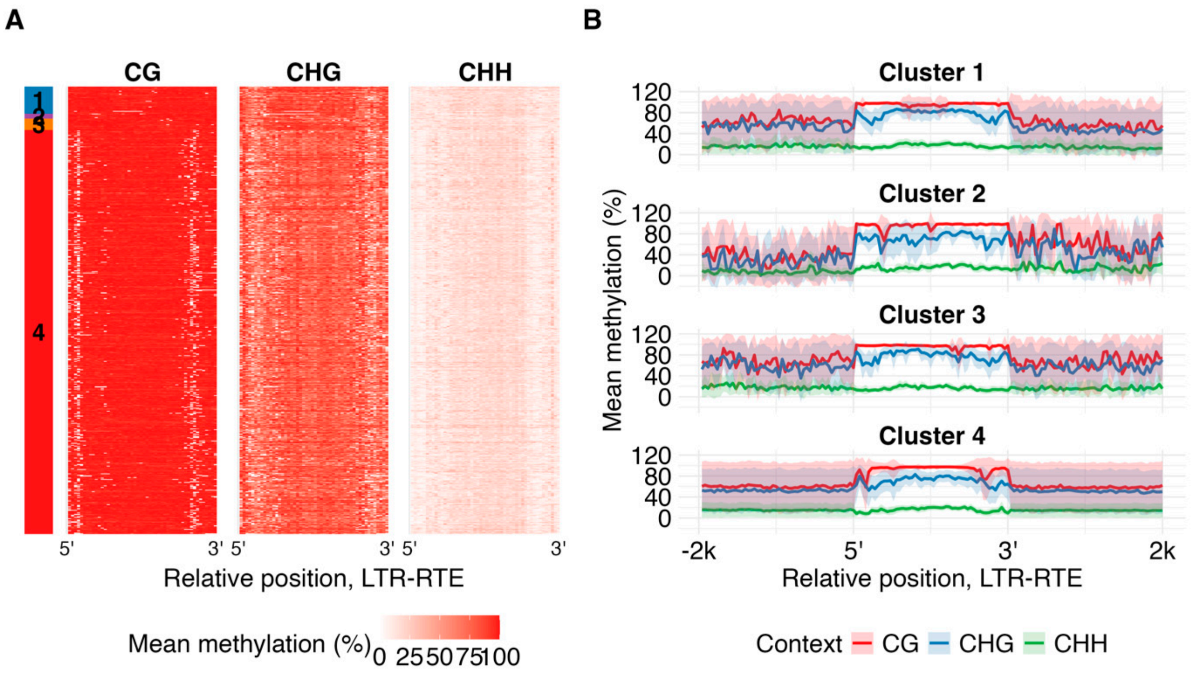

Supplementary Figure S8. Methylation patterns of *H. annuus Tekay* LTR-RTE lineage. (A) Heatplot of LTR-RTEs average methylation level across element length for CG, CHG and

CHH contexts, rows represent individual elements grouped on clusters, where each row corresponds to a leaf on the phylogenetic tree. (B) Methylation pattern of individual clusters which show smoothed mean (solid line) and standard deviation (shaded area) of binned methylation.

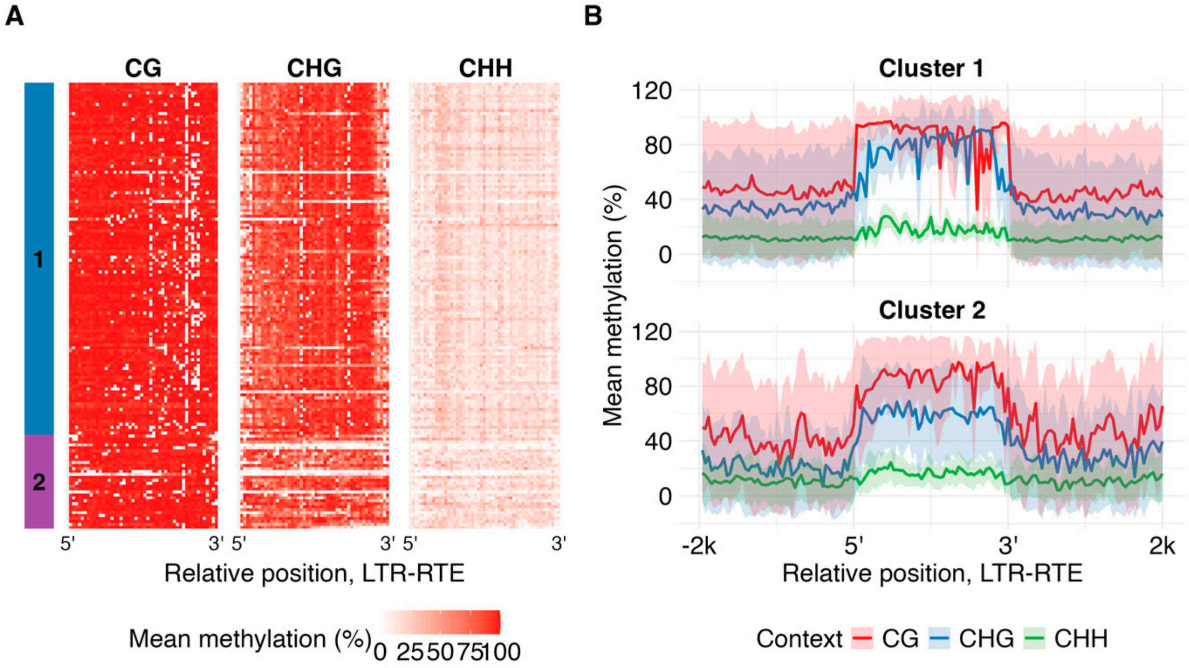

Supplementary Figure S9. Methylation patterns of *H. annuus Ivana* LTR-RTE lineage. (A) Heatplot of LTR-RTEs average methylation level across element length for CG, CHG and CHH contexts, rows represent individual elements grouped on clusters, where each row corresponds to a leaf on the phylogenetic tree. (B) Methylation pattern of individual clusters which show smoothed mean (solid line) and standard deviation (shaded area) of binned methylation.

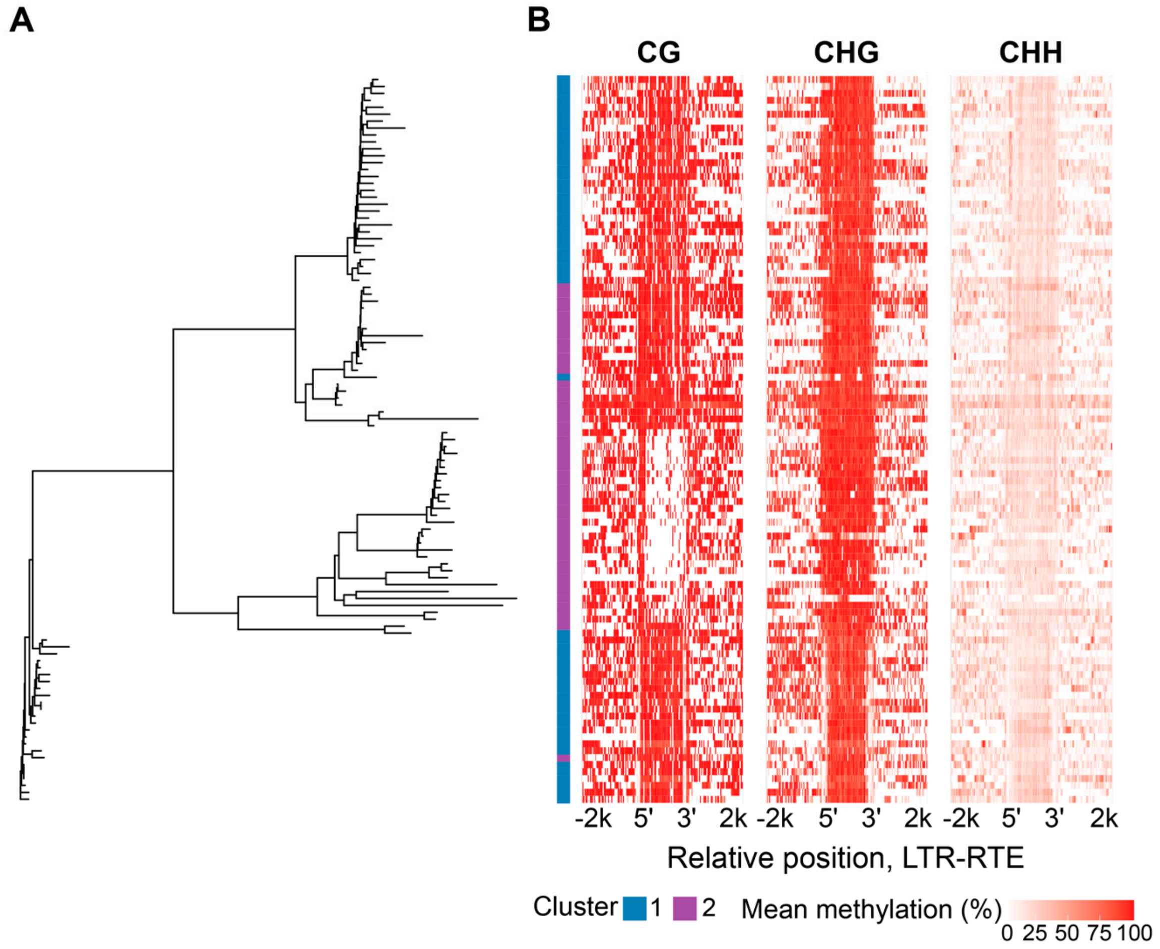

Supplementary Figure S10. Methylation patterns and phylogeny of *H. annuus Tork* LTR-RTE lineage. (A) Phylogenetic tree of reverse transcriptases from individual elements, constructed using the neighbor-joining method. (B) Heatplot of LTR-RTEs average methylation level across element length for CG, CHG and CHH contexts, rows represent individual elements grouped on clusters, where each row corresponds to a leaf on the phylogenetic tree. (C) Methylation pattern of individual clusters which show smoothed mean (solid line) and standard deviation (shaded area) of binned methylation.

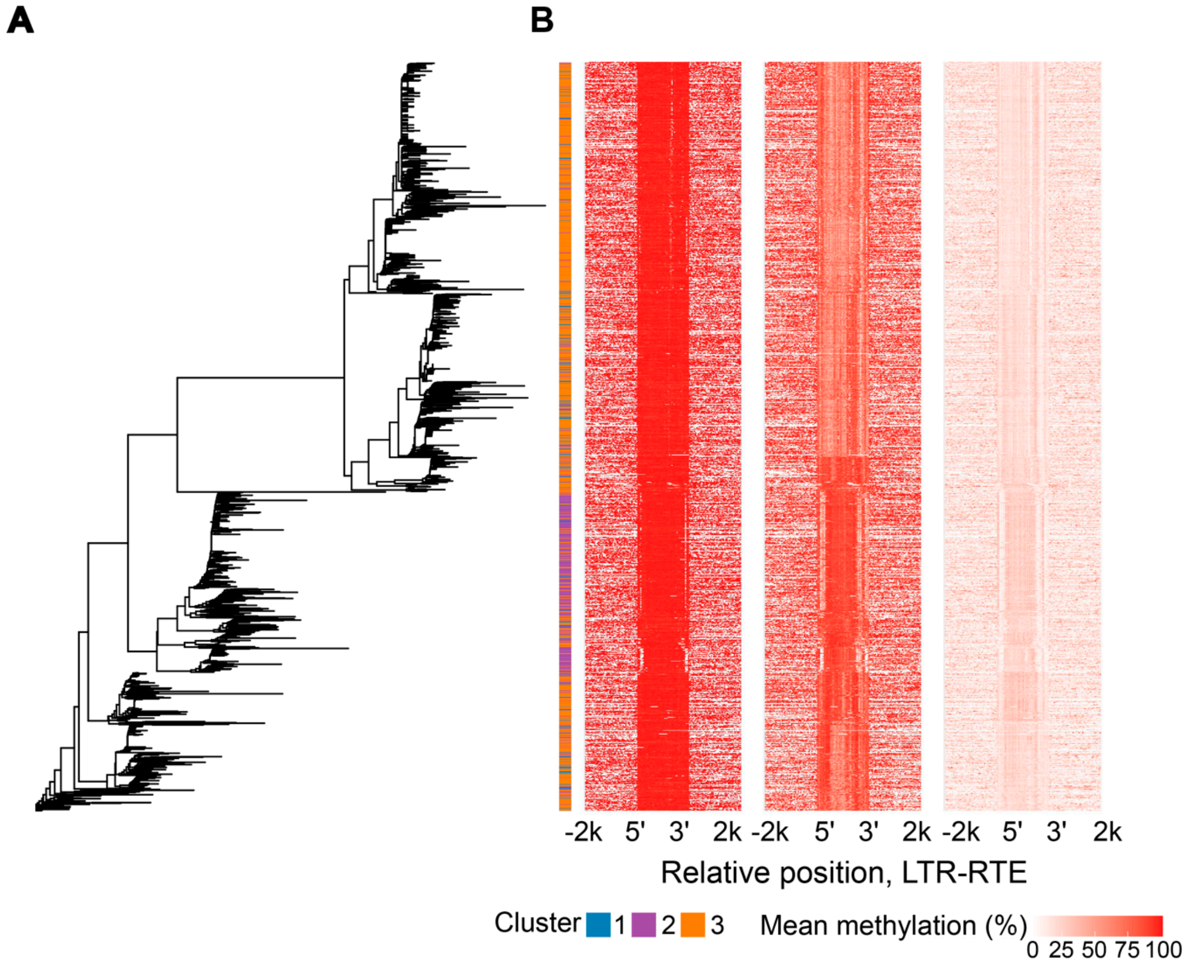

Supplementary Figure S11. Methylation patterns and phylogeny of *H. annuus Athila* LTR-RTE lineage. (A) Phylogenetic tree of reverse transcriptases from individual elements, constructed using the neighbor-joining method. (B) Heatplot of LTR-RTEs average methylation level across element length for CG, CHG and CHH contexts, rows represent individual elements grouped on clusters, where each row corresponds to a leaf on the phylogenetic tree.

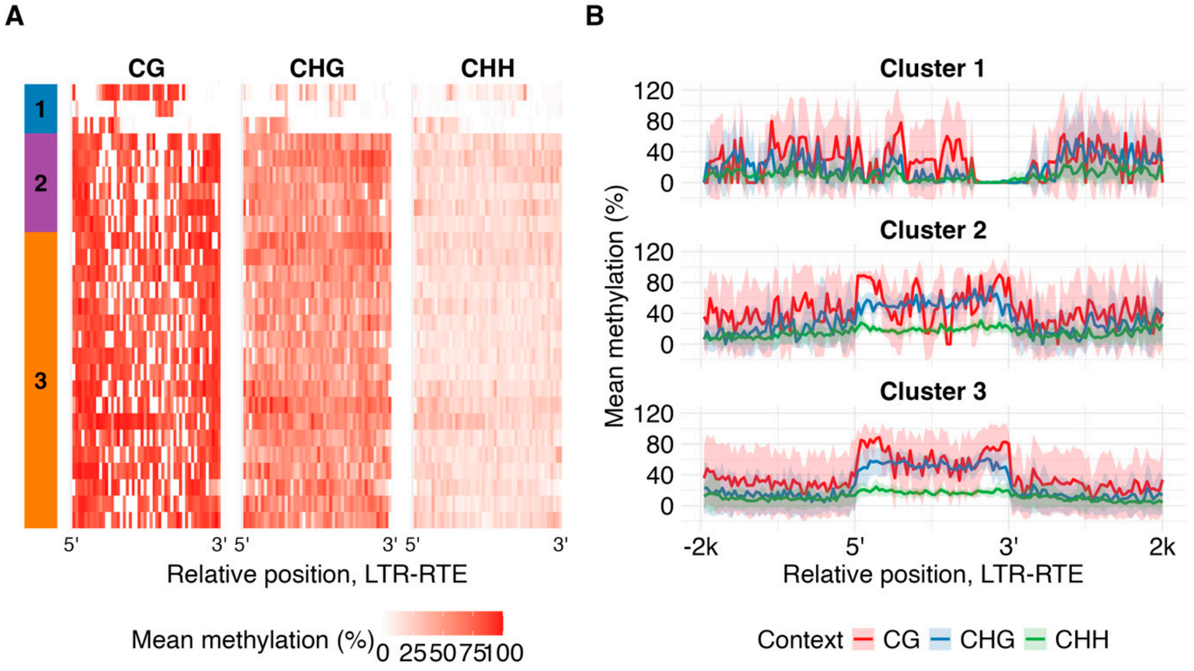

Supplementary Figure S12. Methylation patterns of *A. thaliana Tork* LTR-RTE lineage. (A) Heatplot of LTR-RTEs average methylation level across element length for CG, CHG and CHH contexts, rows represent individual elements grouped on clusters, where each row corresponds to a leaf on the phylogenetic tree. (B) Methylation pattern of individual clusters which show smoothed mean (solid line) and standard deviation (shaded area) of binned methylation.

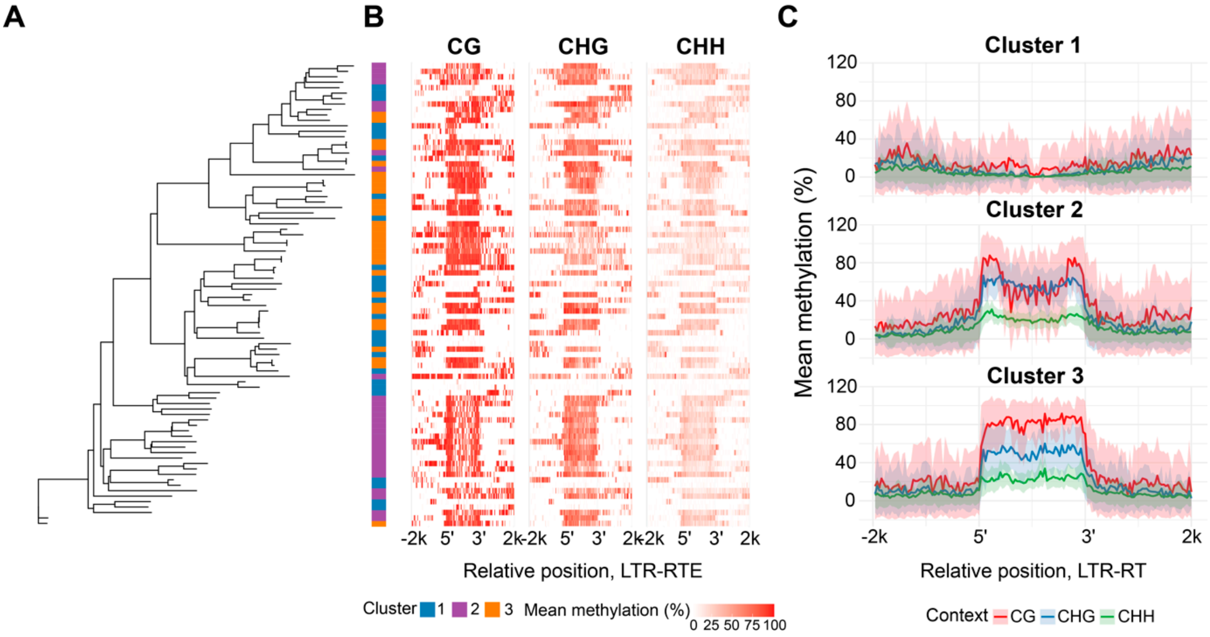

Supplementary Figure S13. Methylation patterns and phylogeny of *A. thaliana Ale* LTR-RTE lineage. (A) Phylogenetic tree of reverse transcriptases from individual elements, constructed

using the neighbor-joining method. (B) Heatplot of LTR-RTEs average methylation level across element length for CG, CHG and CHH contexts, rows represent individual elements grouped on clusters, where each row corresponds to a leaf on the phylogenetic tree.

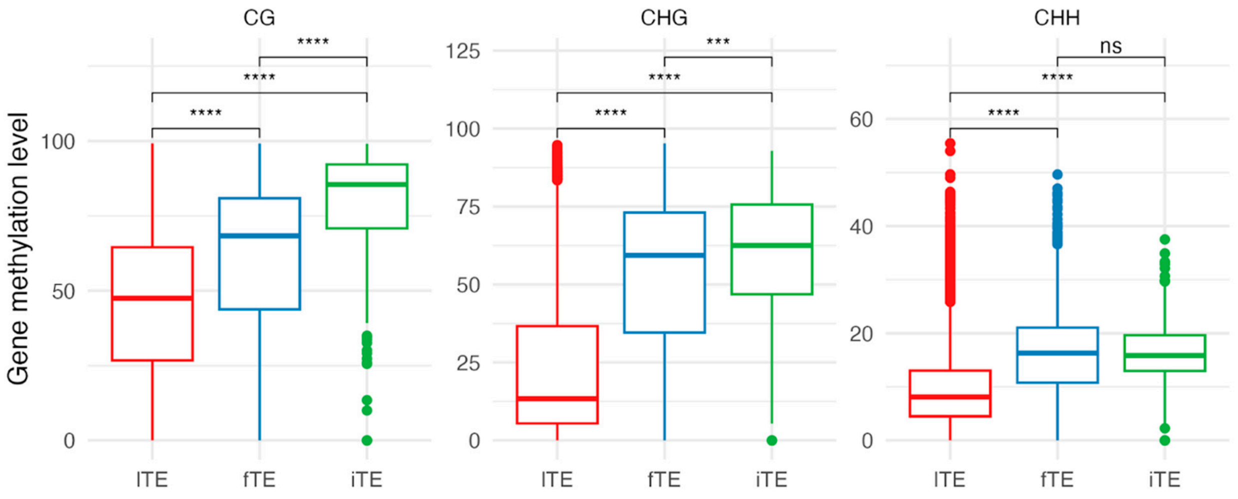

Supplementary Figure S14. Gene methylation level across genes harbouring fragmented TE insertions (fTE), intact TE insertions (iTE) and genes lacking TE insertions (lTE), where "***", "****" and "ns" referred to *p*-value < 0.05, *p*-value < 0.01 and no significance, respectively.

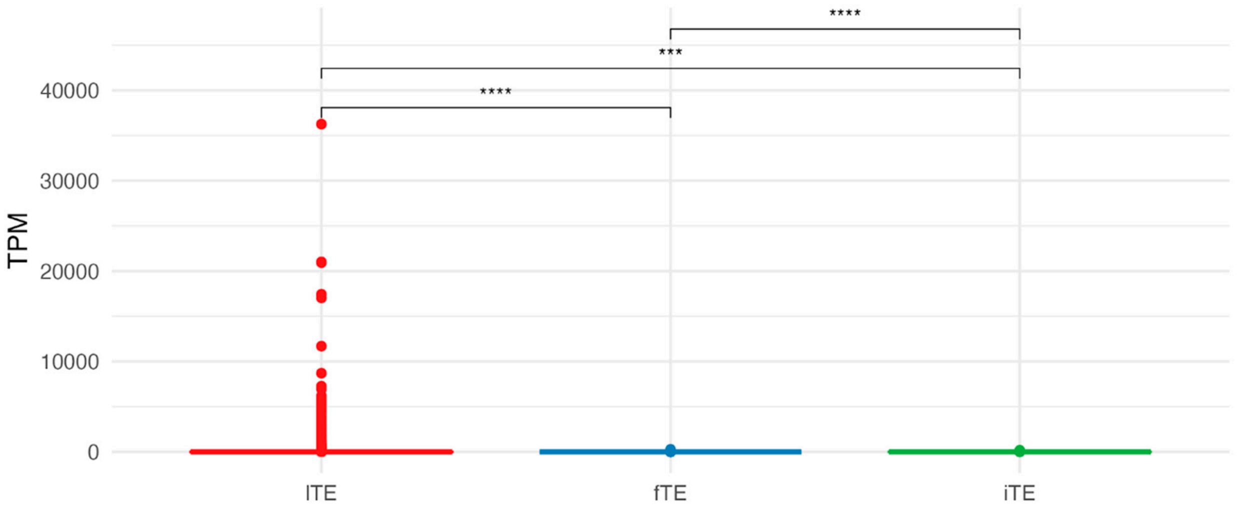

Supplementary Figure S15. Gene expression in TPM units across genes harbouring fragmented TE insertions (fTE), intact TE insertions (iTE) and genes lacking TE insertions (lTE), where "***" and "****" referred to *p*-value < 0.05 and *p*-value < 0.01, respectively

.

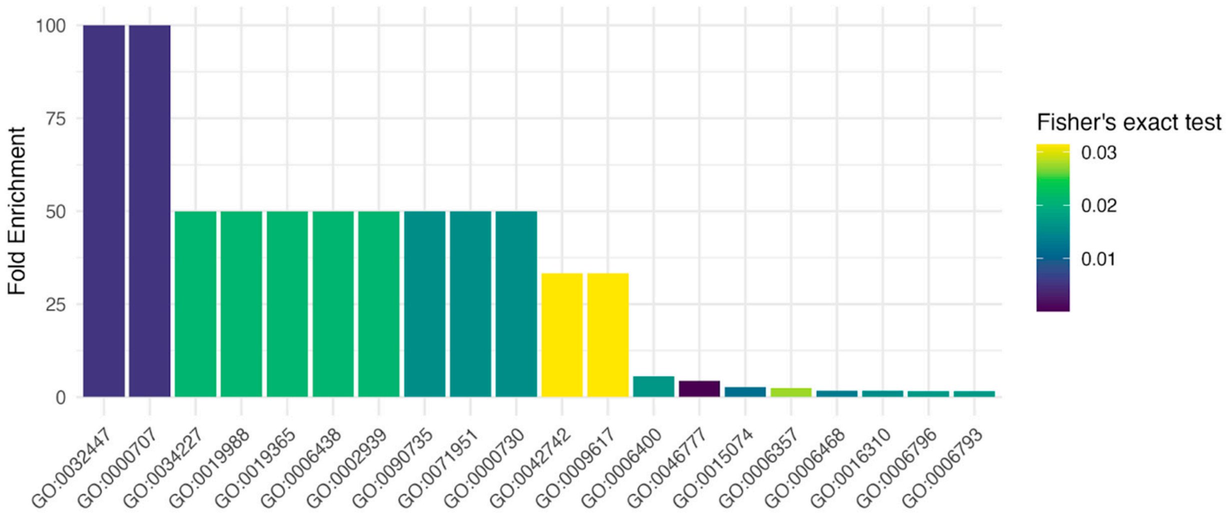

Supplementary Figure S16. Gene ontology (GO) analysis for genes harboring intact TE insertions (iTE).

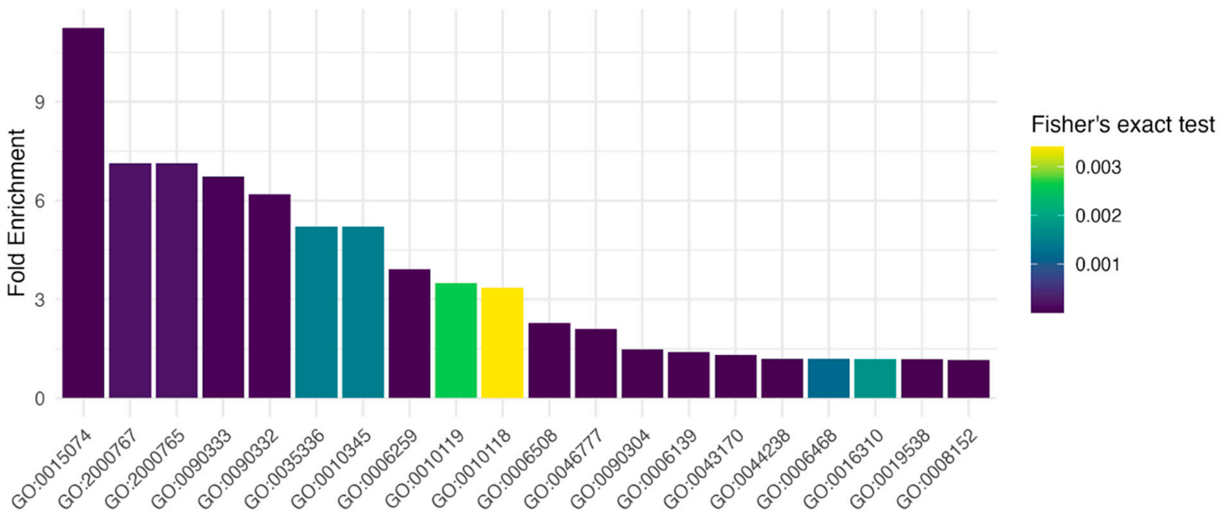

Supplementary Figure S17. Gene ontology (GO) analysis for genes harboring fragmented TE insertions (fTE).

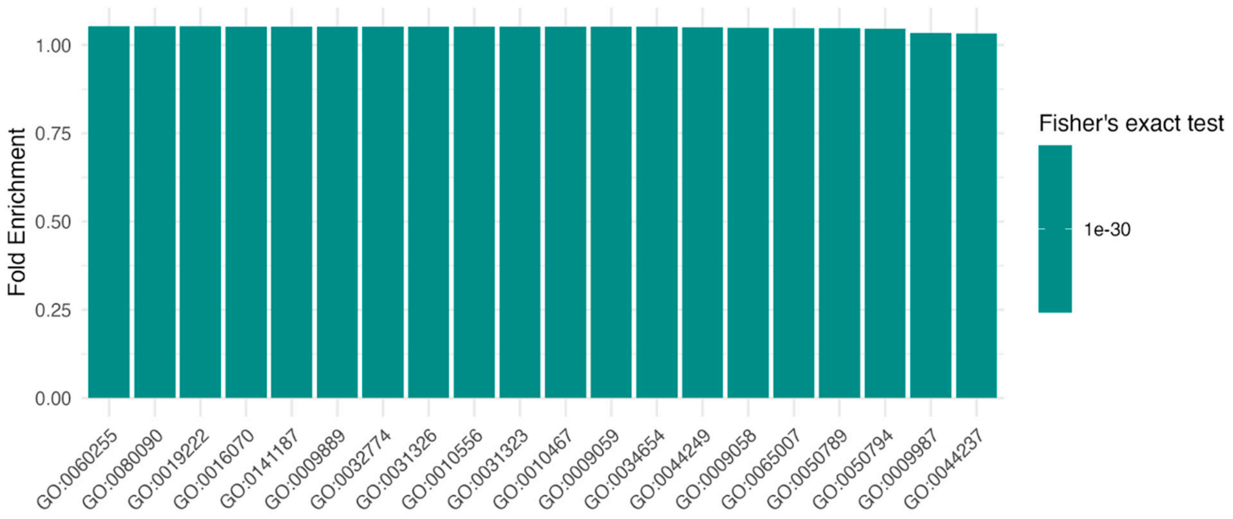

Supplementary Figure S18. Gene ontology (GO) analysis for genes lacking TE insertions (lTE).
